# Cellular Regulation of Kynurenic Acid-Induced Cell Apoptosis Pathways in AGS Cells

**DOI:** 10.3390/ijms23168894

**Published:** 2022-08-10

**Authors:** Hun Hwan Kim, Se Hyo Jeong, Sang Eun Ha, Min Yeong Park, Pritam Bhagwan Bhosale, Abuyaseer Abusaliya, Chung Kil Won, Jeong Doo Heo, Hyun Wook Kim, Gon Sup Kim

**Affiliations:** 1 Research Institute of Life Science, College of Veterinary Medicine, Gyeongsang National University, Jinju 52828, Korea; 2 Biological Resources Research Group, Gyeongnam Department of Environment Toxicology and Chemistry, Korea Institute of Toxicology, 17 Jegok-gil, Jinju 52834, Korea; 3 Division of Animal Bioscience & Integrated Biotechnology, Jinju 52725, Korea

**Keywords:** kynurenic acid, apoptosis, gastric cancer, phenolic compound, treatment

## Abstract

Kynurenic acid was included in the three compounds (caffeic acid, chlorogenic acid, and kynurenic acid) that showed high antioxidant and anti-inflammatory potential among the phenolic compounds contained in *Gynura procumbens*. In this study, the mechanism of cancer cell death induced by kynurenic acid (KYNA), which has the highest molecular binding affinity, in the gastric cancer cell line AGS was confirmed in molecular docking analysis. KYNA showed the most cancer cell death effect on AGS cells among several gastric cancer cell lines (MKN, AGS, and SNU). AGS cells were used for later experiments, and KYNA concentrations of 0, 150, 200, and 250 µM were used. KYNA inhibited cell migration and proliferation in AGS cells in a concentration-dependent manner. G2/M phase cell cycle arrest and reduction of related proteins (Cdc25C, CDK1 and CyclinB1) were confirmed in KYNA-treated AGS cells. Apoptosis of KYNA-treated AGS cells was confirmed by Annexin V/propidium iodide (PI) staining flow cytometry analysis. As a result of morphological chromatin condensation through DAPI (4′,6-diamidino-2-phenylindole), intense blue fluorescence was confirmed. The mechanism of apoptosis induction of KYNA-treated AGS cells was confirmed by western blotting. In the extrinsic pathway, apoptosis induction markers FasL, Fas, and Caspase-3 and -8 were increased in a concentration-dependent manner upon KYNA treatment. In the intrinsic pathway, the expression of anti-apoptotic factors PI3K, AKT, and Bcl-xL was down-regulated, and the expression of apoptosis-inducing factors BAD, Bak, Bax, Cytochrom C, and Caspase-9 was up-regulated. Therefore, in the present study, we strongly imply that KYNA induces apoptosis in AGS gastric cancer cells. This suggests that KYNA, a natural compound, could be the basis for drug for the treatment of gastric cancer.

## 1. Introduction

Stomach cancer is common with a high incidence rate, and is known to be usually caused by *Helicobacter pylori* infection [1]. Two-dimensional factors include irregular eating habits, obesity, fermented dairy products, smoking, and alcohol consumption that influence the development of gastric cancer [2]. Although gastric cancer is usually treated through surgical procedures, the prognosis is poor, and the risk of recurrence is high [3]. Since the treatment of chemotherapeutic agents causes numerous side effects, it is essential to review the drugs used for treatment and to alleviate the harmful effects of treatment [4]. For this reason, it is necessary to develop new preventive and therapeutic agents with low toxicity and excellent effects to replace chemical drugs with a high risk of side effects.

Polyphenols are found in a variety of natural plant sources and are organic components with two or more phenolic units [5]. Polyphenols are produced as secondary metabolites of plants due to their self-protection properties, and are abundantly contained in seeds, leaves, stems, and fruits of plants [6]. Polyphenols are known to have physiological activities such as antioxidant, anti-inflammatory, antiviral, antibacterial, and anticancer [7]. The polyphenols inhibit cancer cell proliferation, eliminate cancer cells through signaling pathways, and contribute to various mechanisms that induce cell cycle and apoptosis [8]. In addition, the intake of polyphenols from diet and supplements has been reported to be safe with low biological and pharmacological side effects [9].

One of several types of cell death, apoptosis consists of two major pathways: the intrinsic apoptosis pathway, initiated within the mitochondria, and the extrinsic apoptosis pathway, initiated by the death ligand-receptor system [10,11]. Both pathways activate caspase-3, leading to cleavage of the sub-factor polymer adenosine diphosphate ribose [12]. One of the major characteristics of tumor cells, the indiscriminate growth and proliferation of cells, is manifested by the imbalance and deregulation of cell cycle regulators [13]. One of the endogenous pathways, the PI3K/AKT signaling pathway, regulates cell growth and metabolism, and the reduction of key factors is known to induce tumor cell death [14]. Polyphenols can be applied as effective therapeutic and prophylactic agents through various anticancer mechanisms by inhibiting cell growth and proliferation through intrinsic and extrinsic pathways [15].

Kynurenic acid (KYNA) is one of the tryptophan catabolic metabolites formed through the kynurenine metabolic pathway, known as a neuroprotective agent in the central nervous system, and is present in a variety of plants. It has been reported that kynurenic acid inhibits cell proliferation and growth by regulating PI3K/AKT and MAPK signaling pathways in adenocarcinoma [16,17]. It is also known to have anticancer effects on kidney cancer cells [18]. However, the apoptosis mechanism and signaling pathway of KYNA for gastric cancer cell line AGS cells have not been studied in detail to date. Therefore, in this study, the apoptosis mechanism and signaling pathway for KYNA in AGS cells, a gastric cancer cell line, were investigated.

## 2. Results

### 2.1. Prediction of the Binding of TOP1 with the Top Three Compounds Using Molecular Docking Analysis

**Figure 1:**
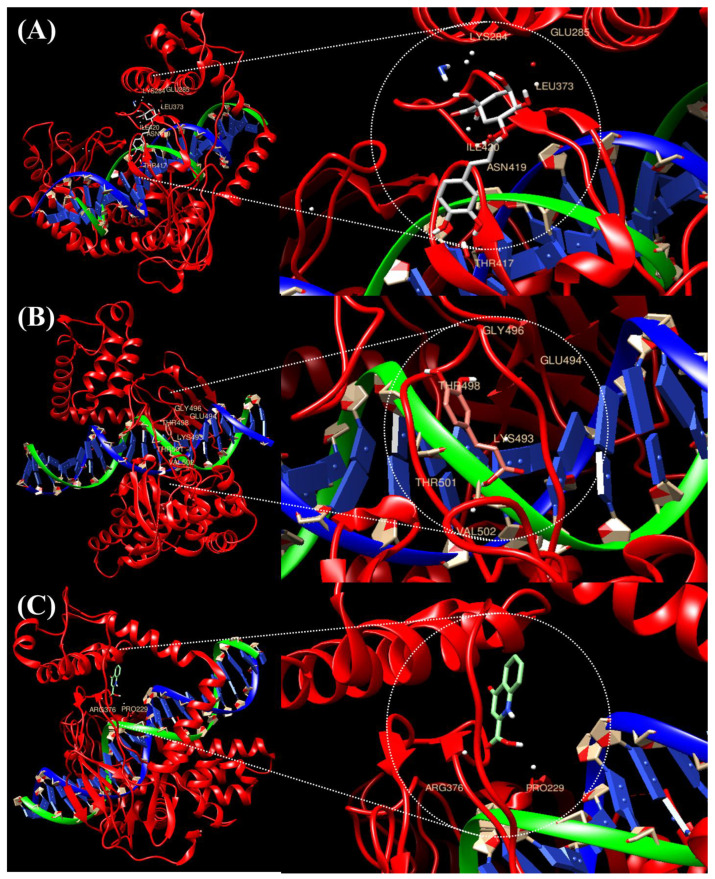
**Molecular docking analysis of the ligands caffeic acid, chlorogenic acid, and kynurenic acid with target TOP1.** The 3D structure of protein TOP1 bound efficiently with the compound caffeic acid (**A**), chlorogenic acid (**B**), and kynurenic acid (**C**) as shown in their interacting amino acids.

In silico molecular docking of the three compounds (caffeic acid, chlorogenic acid and kynurenic acid) with antioxidant and anti-inflammatory potential and TOP1, which is known to be a target for cancer treatment, compared the three compounds through their binding affinity. Ligand-protein docking was performed using UCSF Chimera software (https://www.cgl.ucsf.edu/chimera accessed on 23 May 2022). Figure 1 and Table 1 present the results, which show the bound complex of TOP1 and the three compounds. In the molecular docking score, caffeic acid showed the lowest free energy of −6.1 kcal/mol, followed by chlorogenic acid at −6.4 kcal/mol. The free energy of kynurenic acid and TOP1 was the highest at −6.6 kcal/mol, and the interacting amino acid residues involved in the bound complex were PRO229 and ARG376.

### 2.2. KYNA Induces Cytotoxicity of Gastric Cancer Cells

**Figure 2:**
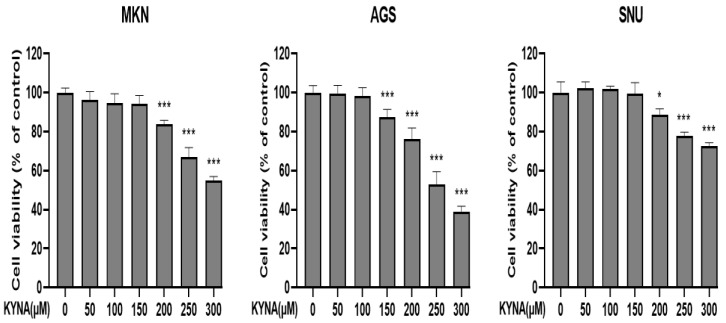
**Effect of KYNA on cell viability of gastric cancer cells.** The inhibition of cell viability was measured by a 3-(4,5-dimethylthiazol-2-yl)-2,5 diphenyltetrazolium bromide (MTT) assay with various concentrations (0, 50, 100, 150, 200, 250 and 300 μM) of KYNA for 24 h. Cell viability is represented in percentage relative absorbance compared to the controls. The results obtained from three independent experiments were expressed as mean ± standard error of the mean (SEM) compared with the control group. * *p* < 0.05, *** *p* < 0.001.

Gastric cancer cell lines MKN, AGS, and SNU cells were treated with or without KYNA (0, 50, 100, 150, 200, 250, and 300 μM) at various concentrations for 24 h. As shown in Figure 2, KYNA showed a concentration-dependent inhibitory effect on all cell lines compared to the control group at 24 h. Among them, the cell line showing the most sensitive effect was AGS cells, which was estimated at a 50% inhibitory concentration of 250 μM. Therefore, AGS cell lines were selected to consider KYNA at concentrations of 0, 150, 200, and 250 μM for further experiments. The cytotoxic effect of KYNA has been demonstrated in AGS cells.

### 2.3. KYNA Inhibits Cell Migration and Proliferation of AGS Cells

**Figure 3:**
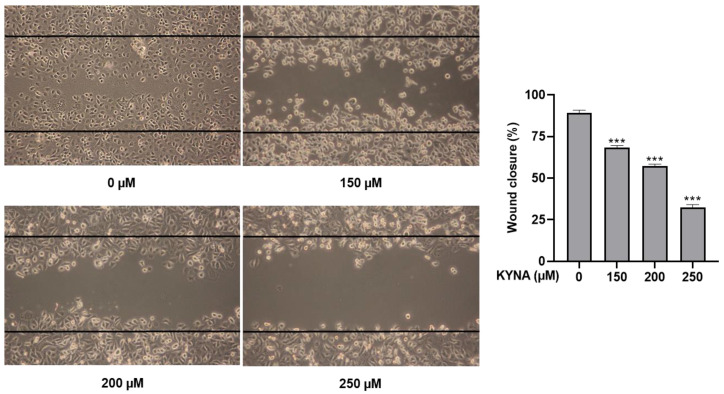
**Effect of KYNA on cell migration of AGS cells.** Wound-healing assay measured the migration ability changes. Cells were treated with KYNA (0, 150, 200, and 250 μM) for 24 h. The results obtained from three independent experiments were expressed as mean ± standard deviation (SD) compared with the control group. *** *p* < 0.001.

**Figure 4:**
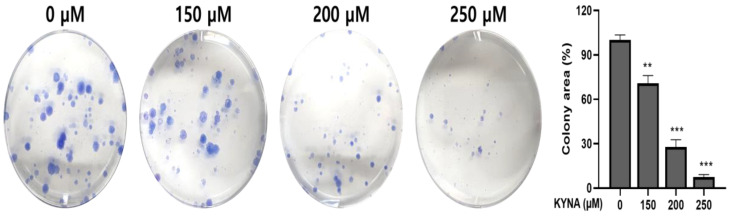
**Effect of KYNA on cell proliferation of AGS cells.** Colony formation assay measured the anti-proliferation effect. Cells were treated with KYNA (0, 150, 200, and 250 μM) for 2 weeks. The results obtained from three independent experiments were expressed as mean ± standard deviation (SD) compared with the control group. ** *p* < 0.01, *** *p* < 0.001.

KYNA was treated at the indicated concentrations (0, 150, 200 and 250 μM) for 24 h to determine the effect of inhibiting proliferation and migration in AGS cells. Inhibition of cell migration was confirmed by a wound healing assay, and, as shown in Figure 3, KYNA significantly inhibited cell migration in a concentration-dependent manner. The result of cell proliferation confirmed by the colony formation assay also confirmed that the area of colonies decreased in a concentration-dependent manner (Figure 4).

### 2.4. KYNA Induces Cell Cycle Arrest of AGS Cells

**Figure 5:**
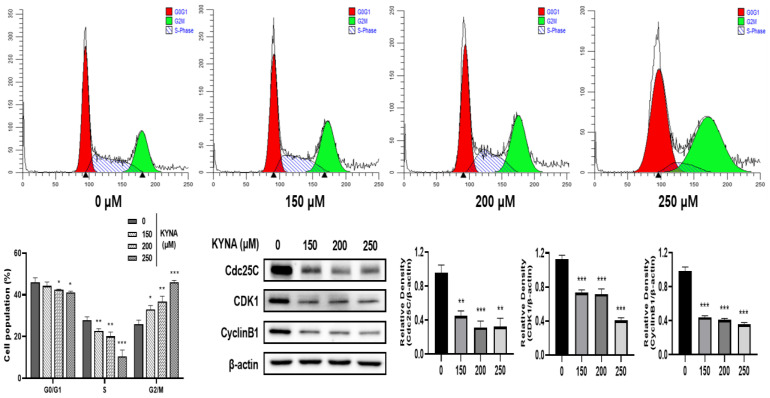
**Effect of KYNA on cell cycle arrest of AGS cells.** KYNA induces AGS cell G2/M cell cycle arrest. Cells were treated with KYNA (0, 150, 200, and 250 μM) for 24 h, and the cell cycle status was detected by flow cytometry. The results obtained from three independent experiments were expressed as mean ± standard deviation (SD) compared with the control group. * *p* < 0.05, ** *p* < 0.01, *** *p* < 0.001.

To investigate the effect of KYNA on the cell cycle arrest of AGS cells, flow cytometry was performed. Cells were treated with KYNA at the indicated concentrations (0, 150, 200, and 250 μM) for 24 h, and then stained with propidium iodide (PI) to analyze the cell cycle (Figure 5). KYNA increased the ratio of cell cycle G2/M phase in a concentration-dependent manner. In addition, the expression levels of Cdc25C, CDK1, and CyclinB1, which are related proteins on G2/M, were significantly reduced. Therefore, it was confirmed that KYNA induces arrest in G2/M phase in AGS cells.

### 2.5. KYNA Induces Apoptosis and Affects the Nuclear Morphology of AGS Cells

**Figure 6:**
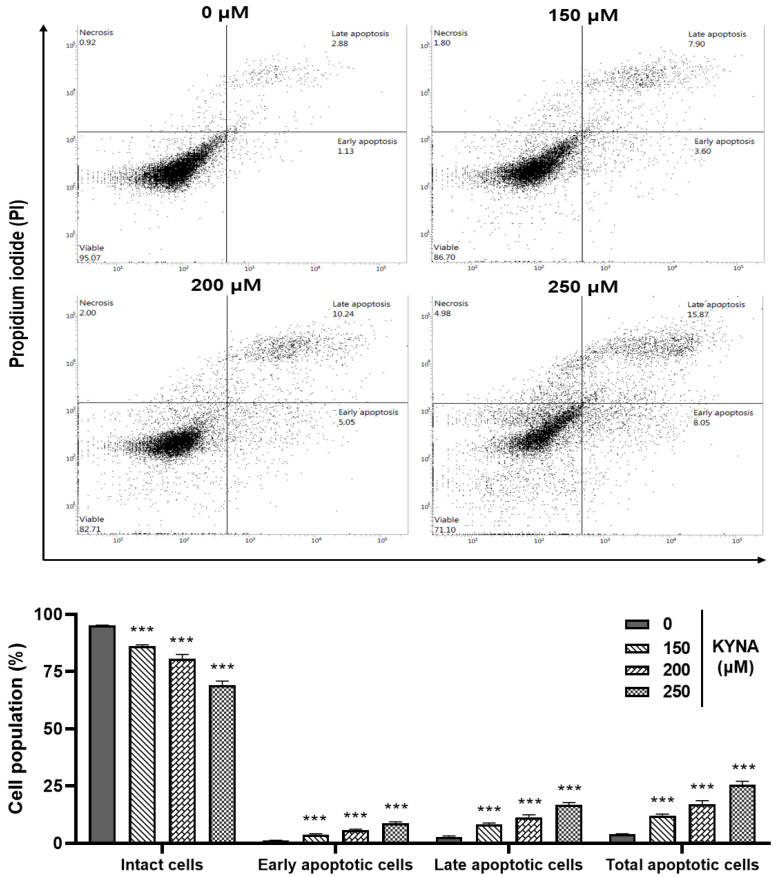
**Effect of KYNA on apoptosis in AGS cells.** To quantify the extent of KYNA-induced apoptosis, the cells were untreated or treated with KYNA at indicated concentrations (150, 200, and 250 μM) for 24 h. Allophycocyanin(APC)/Annexin V and propidium iodide (PI) double-staining was performed which was analyzed by flow cytometry. The results obtained from three independent experiments were expressed as mean ± standard error of the mean (SEM) compared with the control group. *** *p* < 0.001.

**Figure 7:**
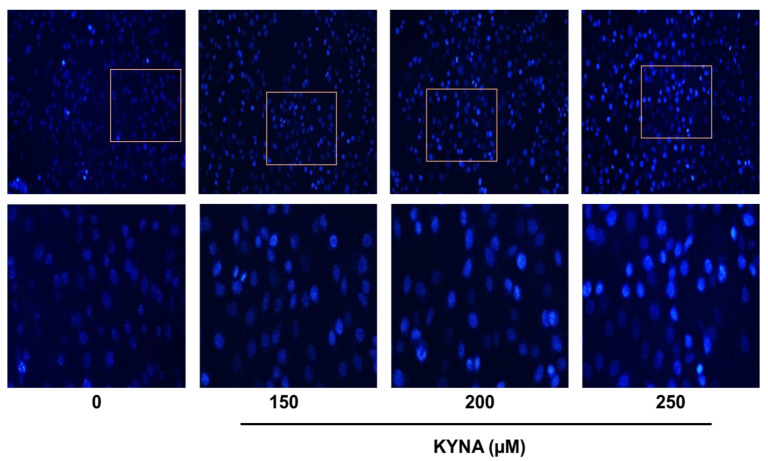
**Nuclear morphological changes on KYNA treated AGS cells.** AGS cells were treated with KYNA at indicated concentrations (150, 200, and 250 μM) for 24 h. After fixation, the cells were stained with 40-6-diamidino-2-phenylindole (DAPI) to observe fragmented chromatin and apoptotic bodies.

The apoptosis-inducing effect of KYNA was confirmed by double staining (Allophycocyanin (APC)/Annexin V and propidium iodide (PI)) flow cytometry. Treatment with 0, 150, 200 and 250 μM of KYNA increased early apoptosis (1.13%, 3.60%, 5.05% and 8.05%) and late apoptosis (2.88%, 7.90%, 10.24% and 15.87%) in a concentration-dependent manner, thereby increasing the total number of apoptotic cells (Figure 6). The nuclear damage effect of KYNA was confirmed through 4′,6-diamidino-2-phenylindole (DAPI) analysis. It was confirmed that the number of chromatin pyknosis displayed in bright fluorescent color and the number of divided nuclei displayed as blue granules increased in a concentration-dependent manner upon KYNA treatment (Figure 7). These results show that the KYNA induced apoptosis in AGS cells.

### 2.6. KYNA Induces Extrinsic Apoptosis Pathway of AGS Cells

**Figure 8:**
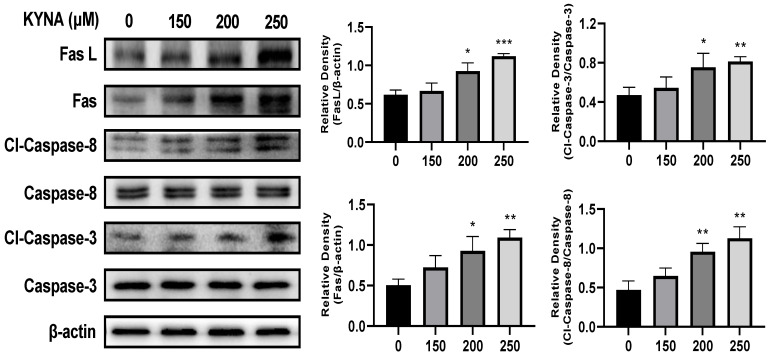
**KYNA induced extrinsic apoptosis pathway of AGS cells.** The cells were treated with KYNA at indicated concentrations (0, 150, 200, and 250 μM) for 24 h. Fas ligand (FasL), Fas, cleaved caspase-8, and cleaved caspase-3 levels were quantified by western blot. The results obtained from three independent experiments were expressed as mean ± standard error of the mean (SEM) compared with the control group. * *p* < 0.05, ** *p* < 0.01, *** *p* < 0.001.

To determine the effect of KYNA on the extrinsic apoptosis pathway of AGS cells, the expression of related proteins was analyzed by using a western blot (Figure 8). Treatment with KYNA up-regulated the expression of FasL and Fas in a concentration-dependent manner, which resulted in the subsequent factors of cleaved caspase-3 and -8, and thus the expression was up-regulated. KYNA treatment in AGS cells significantly increased the expression of apoptosis-inducing factors involved in the extrinsic apoptosis pathway. These results suggest that KYNA induces apoptosis through the extrinsic apoptotic pathway in AGS cells.

### 2.7. KYNA Induces Intrinsic Apoptosis Pathway of AGS Cells

**Figure 9:**
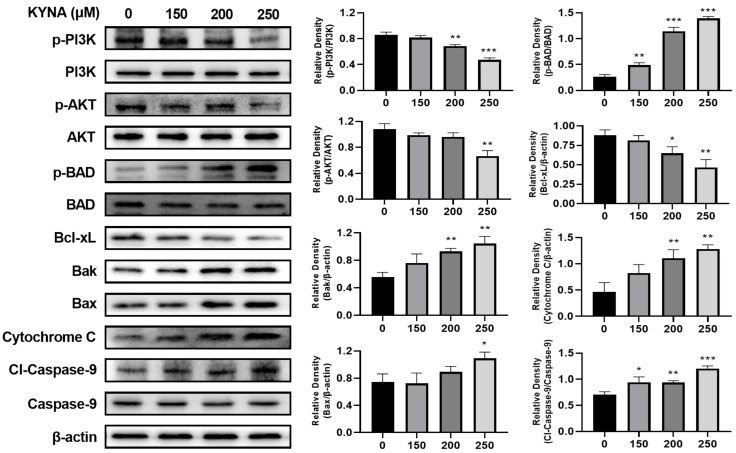
KYNA induced intrinsic apoptosis pathway in AGS cells. The cells were treated with KYNA at indicated concentrations (0, 150, 200, and 250 μM) for 24 h. P-phosphoinositide 3-kinase (p-PI3K), p-protein kinase B (p-AKT), Bcl-2-associated death promoter (BAD), Bcl-xL Bak, Bax, Cytochrome C, and cleaved caspase-9 levels were quantified by western blot. The results obtained from three independent experiments were expressed as mean ± standard error of the mean (SEM) compared with the control group. * *p* < 0.05, ** *p* < 0.01, *** *p* < 0.001.

To investigate the effect of KYNA on the intrinsic apoptosis pathway of AGS cells, the expression of related proteins was confirmed by western blot (Figure 9). Anti-apoptosis factors, p-PI3K and p-AKT, significantly decreased with KYNA treatment. As a result, the expression of p-Bad, an apoptosis factor, was increased. Expression of intrinsic apoptosis factors Bak and Bax increased due to increased expression of cleaved-caspase-8 corresponding to the extrinsic apoptosis pathway, and increased expression of Bad decreased the expression of Bcl-xL. Bak, Bax, and Bcl-xL upregulated the expression of cytochrome C, an apoptosis factor. The up-regulated expression of cytochrome C induces the expression of cleaved-caspase-9, which induces the cleavage of caspase-3. KYNA treatment in AGS cells induced a decrease in the expression of anti-apoptosis factors corresponding to the intrinsic apoptosis pathway and an increase in the expression of apoptosis factors. These results suggest that KYNA induces the intrinsic apoptosis pathway.

## 3. Discussion

Gastric cancer is a common tumor occurring in the gastrointestinal tract and is the second leading cause of death worldwide. Research is being conducted to develop therapeutic agents to prevent and treat it [19]. Among them, polyphenols, which are abundant in plant materials, have no side effects compared to chemical drugs; they inhibit the growth and proliferation of tumor cells, thereby interfering with various cancer mechanisms, such as cell cycle regulation and apoptosis, and effectively show anticancer effects [20]. Tumor apoptotic effects on lung, breast, and colon cancers have been reported [21], and a wide range of properties such as anti-angiogenesis, anti-metastatic, and DNA interactions suggest the potential of polyphenols [22].

In previous studies, three compounds with antioxidant and anti-inflammatory potential were selected [23], and kynurenic acid (KYNA), which has the highest molecular binding affinity through molecular docking analysis, was used for future analysis. KYNA is a physiologically active ingredient found in various herbs [24], and is known to induce apoptosis through cell cycle regulation and signal transduction pathways in colon cancer cells [18]. As a result of analyzing the apoptosis effect of KYNA on gastric cancer cell lines and MKN, AGS, and SNU cells, the most effective apoptosis effect on AGS cells was confirmed (Figure 2). Therefore, the anticancer effect of three fixed doses of KYNA in AGS cells was investigated. First, as a result of confirming the anti-migration and anti-proliferative effects of KYNA on AGS cells, the migration and proliferation of AGS cells were significantly inhibited, as shown in Figure 3 and Figure 4.

In normal cells, replication and apoptosis through the cell cycle are balanced, but in tumor cells, this balance is disrupted [25]. By targeting cyclins and cyclin-dependent kinases (CDKs) that regulate the cell cycle, it can be investigated whether they can affect cell proliferation, and an increase in cell number in the G2/M phase is associated with apoptosis [26]. As a result of confirming the effect of KYNA on the cell cycle of AGS cells, it was confirmed that the number of cells in G2/M phase was increased, and the expression of related protein factors decreased (Figure 5). This is because KYNA decreased the expression of cyclin B1 protein. It induces the arrest of G2/M phase and suppresses the transcription of cyclin B1 to regulate the cell cycle, suggesting that AGS cell death is induced [27].

Control of apoptosis in tumor cells is an effective cancer treatment, requiring an understanding of various apoptosis mechanisms [28]. Malignant cells have the characteristic of trying to avoid apoptosis, and induction of apoptosis is the most promising method for treating tumors [29]. Among them, apoptosis, a type of programmed cell death, is a representative mechanism involved in tumor cell death [30]. In this study, it was confirmed whether KYNA induces apoptosis in AGS cells through APC/V and PI double staining. KYNA increased early and late apoptotic cells in a concentration-dependent manner in AGS cells (Figure 6), and DNA condensation characteristic of apoptosis was also confirmed through DAPI staining (Figure 7).

**Figure 10:**
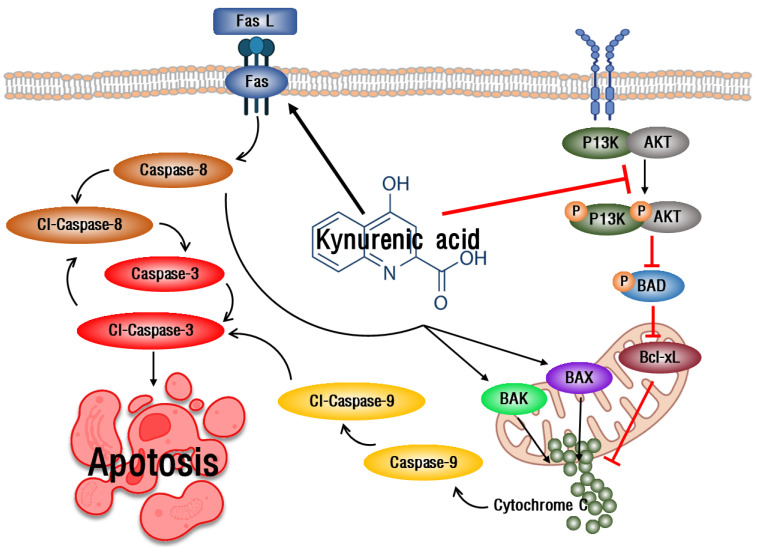
Schematic representation of extrinsic and intrinsic pathway mediated apoptosis induction by KYNA.

The extrinsic apoptosis pathway of apoptosis starts when it binds with apoptosis receptors and the death induction signal complex (DISC), and a representative Fas-mediated apoptosis pathway is used [31]. Fas is known to induce apoptosis by inducing increased expression of caspase-3 and -8, apoptotic factors [32]. When AGS cells were treated with KYNA, the expression of caspase-3 and -8 was increased due to the increase of FasL and Fas, which correspond to the extrinsic apoptosis pathway (Figure 8). This suggests that KYNA induces an extrinsic apoptosis pathway. The PI3K/AKT pathway plays an important role in the migration and growth of cancer cells, and increased expression is known to trigger the development and development of tumor cells [33]. The intrinsic apoptosis pathway is a mitochondrial-dependent apoptosis pathway, which consists of the increased expression of apoptosis-promoting factors Bax and Bak and the decreased expression of anti-apoptotic factor Bcl-xL [34], and promotes apoptosis by inducing the activation of cytochrome C [35]. When AGS cells were treated with KYNA, the expression of PI3K and AKT was decreased and the expression of BAD was increased. It also induced a decrease in the expression of the anti-apoptotic factor Bcl-xL and an increase in the expression of the pro-apoptotic factors Bak, Bax, cytochrome C, and caspase-9 (Figure 9). These results confirmed that KYNA induces extrinsic and intrinsic apoptosis in AGS cells (Figure 10).

## 4. Materials and Methods

### 4.1. Chemicals and Reagents

Kynurenic acid (KYNA) was purchased from Sigma-Aldrich Corp. (St. Louis, MO, USA). 3-(4,5-Dimethylthiazol-2-yl)-2,5-diphenyltetrazolium bromide (MTT) was obtained from Duchefa Biochemie (Haarlem, The Netherlands). Antibodies to caspase-3 (Cat. #9662S, Polyclonal (P)), -8 (Cat. #4790S, Monoclonal (M)), and -9 (Cat. #9502S, P); cleaved caspase-3 (Cat. #9664S, M), -8 (Cat. #9496S, M), and -9 (Cat. #7237S, M); Fas (Cat. #4233S, M); FasL (Cat. #68405S, M); cyclin B1 (Cat. #12231S, M); Cdc25C (Cat. #4688S, M); Bad (Cat. #9292S, P); Cytochrome C (Cat. #4280S, M); p-PI3K (Cat. #4228S, P), PI3K (Cat. #3011S, M); p-AKT (Cat. #4060S, M); AKT (Cat. #4691S, M); Bcl-xL (Cat. #2764S, M); Bax (Cat. #2774S, P); Bak (Cat. #3814S, P); and β-actin (Cat. #4970S, M) were purchased from Cell Signaling Technology (Danvers, MA, USA). Antibodies cdk1 (Cat. 06-923, P) were purchased from Merck Millipore (Temecula, CA, USA).

### 4.2. Selection of Compounds and Molecular Docking Analysis

In our previous study, we selected three compounds with antioxidant and anti-inflammatory potentials [23]. The binding affinity of the three compounds with the tumor-inducing factor (TOP1) was confirmed. For the execution of molecular docking, the structure of DNA topoisomerase 1 (TOP1) was obtained from a protein data bank (PBD) (https://www.rcsb.org/ accessed on 23 May 2022) with PDB ID 1EJ9 at high resolution, and the three-dimensional structure of the compound caffeic acid (CID; 689043), chlorogenic acid (CID; 1794427) and kynurenic acid (CID; 3845) were obtained from PubChem (https://pubchem.ncbi.nlm.nih.gov/ accessed on 23 May 2022). The protein and ligand were subjected to docking using the USCF Chimera and AutoDock Vina programs, and all the possible conformations were obtained with default parameters. The results were evaluated based on the estimated free energy of binding and total intermolecular energy.

### 4.3. Cell Culture and KYNA Treatment

AGS human gastric cancer cells obtained from the Korea Cell Line Bank (Seoul, South Korea) were cultured in RPMI medium containing 10% fetal bovine serum (FBS) and 1% penicillin/streptomycin (P/S) at 37 °C in a humidified atmosphere of 5% CO_2_. KYNA was prepared with dimethyl sulfoxide (DMSO), stored at −20 °C, and diluted to the required concentration with RPMI medium before use. Cells were DMSO-treated or untreated with the indicated concentration of KYNA for 24 h in a complete medium, and, in all experiments, the final concentration of DMSO used was below 0.1%.

### 4.4. Cell Viability Assay

Cell viability was measured using an MTT assay. Cells were seeded at 1 × 10^4^ cells in a 96-well plate and incubated overnight, followed by treatment with KYNA at the concentrations of 0, 50, 100, 150, 200, 250, and 300 μM for 24 h. After incubation, 10 μL of MTT (0.5 mg/mL) solution was added to each well and incubated for about 3 h at 37 °C. The formazan precipitate formed after incubation was dissolved in 100 μL of DMSO, and the absorbance of the converted dye was measured at a wavelength of 540 nm with a micro-plate reader (BioTek, Winooski, VT, USA). Cell viability was expressed as a percentage of proliferation versus the KYNA untreated group.

### 4.5. Wound Healing Assay

AGS cells were seeded into 6-well plates at 5 × 10^5^ cells per well, incubated overnight to form a confluent cell monolayer, and crossed vertically with a yellow tip in the middle of each well. Plates were then washed with PBS to remove the scraped cells. After treatment with the indicated concentrations of KYNA (0, 150, 200, and 250 μM), they were incubated for 24 h. The images of the scratches were obtained under microscopy.

### 4.6. Colony Formation Assay

AGS cells were seeded into 6-well plates at 200 cells per well. The cells were treated with the indicated concentrations of KYNA (0, 150, 200, and 250 μM), followed by incubation for 2 weeks. After incubation, the grown colonies were fixed with 4% paraformaldehyde for 30 min, then stained with 0.6% Giemsa stain for 30 min, and further washed with tap water to remove excess stain. The obtained colonies were counted using the ImageJ software program (U.S. National Institutes of Health, Bethesda, MD, USA).

### 4.7. Analysis of Cell Cycle Distribution

Flow cytometry was performed to analyze the cell cycle distribution. AGS cells were treated with various concentrations of KYNA (0, 150, 200, and 250 μM) for 24 h at 37 °C. The cells were washed with ice-cold PBS. After incubation, trypsinized cells were collected in a 15 mL conical tube, and the pellet was obtained by centrifugation at 1200 rpm for 4 min. The pellets were washed twice with ice-cold PBS and fixed in 70% ice-cold ethanol for 1 h at −20 °C. The cell suspension was centrifuged further, and the cells were washed in PBS and resuspended in 400 μL of PBS containing 50 μg/mL PI (Sigma-Aldrich, St. Louis, MO, USA) and 50 μg/mL RNase A, followed by incubation in dark conditions for 15 min at room temperature. After incubation, flow cytometry analysis was performed on the cell suspensions, and the data obtained were analyzed using the FACSVerseTM flow cytometer (BD Biosciences, Franklin Lakes, NJ, USA). The acquired FACS data were analyzed using ModFit LT 5.0 software (Verity Software House, Topsham, ME, USA).

### 4.8. Annexin V-Propidium Iodide Apoptosis Detection

Apoptotic cells were detected by using an allophycocyanin (APC)/Annexin V apoptosis detection kit according to the manufacturer’s protocol (BD Biosciences, San Diego, CA, USA). Briefly, cells were plated on 60 mm plates at 4 × 10^5^ cells and then incubated with various concentrations of KYNA (0, 150, 200, and 250 μM) for 24 h. The cells were collected and washed with PBS and resuspended in binding buffer. The cells were stained with APC/Annexin V and propidium iodide (PI) for 15 min at room temperature in the dark, before the addition of binding buffer. Flow cytometry analysis was performed on the cell suspensions and the data obtained were analyzed using a fluorescence-activated cell-sorting machine (FACSVerseTM flow cytometer; BD Biosciences, Franklin Lakes, NJ, USA). In total, 10,000 events per sample were sorted and the data were analyzed using BD FACSuiteTM software (BD Biosciences, Becton & Dickson, Mountain View, CA, USA).

### 4.9. Cell Morphological Change and DAPI Staining

For nuclear morphological analysis, AGS cells were plated on 12-well plates at 1 × 10^5^ cells after treatment with various concentrations of KYNA (0, 150, 200, and 250 μM) at 37 °C for 24 h. The cells were washed with ice-cold PBS and then fixed with 37% formaldehyde (1:4 dilutions with PBS) for 15 min at room temperature. Subsequently, the fixed cells were washed with PBS and stained with a 4′,6-diamidino-2-phenylindole (DAPI; Vectashield H-1500; Vector Laboratories, Burlingame, CA, USA). The nuclear morphology of the cells was examined with fluorescence microscopy (EVOS^®^, Life Technologies, Darmstadt, Germany).

### 4.10. Analysis of Protein Expression by Western Blot

AGS cells were seeded into 60 mm plates at 4 × 10^5^ cells per well and treated with the indicated concentrations of KYNA (0, 150, 200, and 250 μM) for 24 h. Cells were lysed using a radioimmunoprecipitation assay (RIPA) buffer (iNtRON Biotechnology, Seoul, Korea) containing phosphatase and a protease inhibitor cocktail (Thermo Scientific, Rockford, IL, USA). Protein concentrations were determined using a Pierce™ BCA assay (Thermo Fisher Scientific, Rockford, IL, USA). An equal quantity of protein (10 μg) from each sample was electrophoresed on (8–15)% SDS-polyacrylamide gels and transferred to a polyvinylidene difluoride (PVDF) membrane (ATTO Co., Ltd., Tokyo, Japan), and then the membrane was incubated with the primary antibodies followed by a conjugated secondary antibody to peroxidase. The obtained proteins were detected by an electrochemiluminescence (ECL) detection system (Bio-Rad Laboratory, Hercules, CA, USA), and analyzed using the Image Lab 4.1 (Bio-Rad) program. The densitometry readings of the protein bands were normalized by comparison with the expression of β-actin as control, using the ImageJ software program (U.S. National Institutes of Health, Bethesda, MD, USA). Antibodies of CDC25C, CyclinB1, FasL, Fas, Caspase-3, Caspase-8, PI3K, AKT, BAD, Bcl-xL, Bak, Bax, Cytochrome C, Caspase-9, and β-actin were purchased from Cell Signaling Technology (Danvers, MA, USA). Antibodies cdk1 were purchased from Merck Millipore (Temecula, CA, USA).

### 4.11. Statistical Analysis

All the experimental results were expressed as the mean ± standard error of the mean (SEM) of triplicate samples. Significant differences between groups were calculated by one-way factorial analysis of variance (ANOVA), followed by a Bonferroni’s test, and *p* < 0.05 was considered statistically significant. * *p* < 0.05, ** *p* < 0.01, *** *p* < 0.001.

## 5. Conclusions

This study demonstrated the anti-shipping effect of kynurenic acid (KYNA) in AGS cells. These results provided information about the effects of KYNA on gastric cancer cells and provided insight into the use of KYNA in the development of drugs for the prevention and treatment of gastric cancer. However, this study has a limitation in that only one among various gastric cancer cell lines was used. Thus, further studies are needed in the future concerning the in vitro analysis performed in the current study.

## Figures and Tables

**Table 1:** Molecular docking analysis of the ligands caffeic acid, chlorogenic acid and kynurenic acid with target TOP1.

Compound-Protein	Interacting Amino Acid Residues	Final Intermolecular Energy
Caffeic acid	GLY496, GLU494, THR498, LYS493, THR501, VAL502	−6.1 kcal/mol
Chlorogenic acid	LYS284, GLU285, LEU373, ILE420, ASN419, THR417	−6.4 kcal/mol
Kynurenic acid	PRO229, AGR376	−6.6 kcal/mol

The table shows the list of interacting amino acids and binding energy.

## Data Availability

The data used to support the findings of this study are available upon request from the corresponding author.
